# Supplementary material for: Ultrasound-guided internal branch of superior laryngeal nerve block on postoperative sore throat: A randomized controlled trial
Source: PLoS One. 2020 Nov 20;15(11):e0241834. doi: 10.1371/journal.pone.0241834 (PMC7679017; doi:10.1371/journal.pone.0241834)
Supplement: S1 Protocol — (DOC) [file pone.0241834.s004.doc]

**喉上神经阻滞治疗术后咽喉痛项目研究方案**

1. **立项依据**

**（一）项目研究的意义和目标**

气管插管是全身麻醉术后导致的口咽喉部或呼吸道黏膜损伤是引起术后咽喉痛 ( **postoperative sore throat,** POST) 的主要原因，本课题主要研究目的是探索治疗全麻拔管后POST更有效的方式，即超声可视引导下喉上神经内侧支阻滞在治疗全麻拔管后咽喉疼痛的应用研究，为降低全身麻醉拔管后POST 的发生率提供参考。项目的主要目标：通过此项目的开展与研究，为气管插管全身麻醉拔管后咽喉痛的治疗提供较好的镇痛方案，提高围手术期患者舒适化程度和满意度，推动学科领域的发展。

**（二）国内外同类产品和技术现状**

术后咽喉痛（POST）是气管插管全麻术后常见的并发症之一，研究表明全身麻醉患者拔管后咽喉痛的发生率达30%～70% ，主要与咽喉部或呼吸道黏膜损伤有关。虽然POST有自愈性，但其会增加患者不适，延长患者住院时间，故仍被患者视为全身麻醉后不满意的不良反应之一。POST 的发生与多方面因素有关，例如气管导管因素，包括其型号、套囊压力； 操作因素，插管时气管粘膜损伤等；手术因素，包括手术时间、手术体位、手术种类等；另外，女性和既往有吸烟史也是POST的高危因素。随着无痛化、舒适化医疗模式及快速康复理念的深入，医务工作者越来越重视 POST的防治，对于接受全身麻醉手术的患者，POST仍是需要解决的重要问题。

传统的处理方法包括药物治疗、早期雾化吸入及气管导管前端处涂抹局麻药物乳膏等，虽然可以缓解一部分POST，但并不能获得预期的理想效果，尤其是在疼痛比较剧烈的患者。有研究表明，双侧喉上神经阻滞可有效抑制拔管期的时应激反应，减少气管拔管和麻醉苏醒期心血管不良事件的发生。喉上神经内支穿甲状舌骨膜入喉，分成许多小支至咽、会厌、梨状隐窝以及声门裂以上的喉黏膜，阻滞喉上神经内支，可达到舌根、 会厌及声门裂以上喉黏膜的麻醉效果。有研究喉上神经阻滞在纤维支气管镜检查、喉内窥镜手术、困难气道清醒插管及辅助插管全麻术中能较好地抑制对气道刺激引起的血流动力学改变，为操作提供较好的条件。国外研究双边喉上神经的内支阻滞辅助喉内窥镜手术的应用效果，并评估对全身麻醉术后咽喉痛的发生率和严重程度的影响，表明喉上神经的内支阻滞有缓解术后咽痛的发生率，减轻术后咽痛的程度，但在单独用于术后咽喉疼痛治疗的研究方面不多见，国内未见相关报道。

**（三）发展趋势和前景预测**

喉上神经的内支阻滞对术后咽喉疼痛治疗效果理想，与传统的方法药物治疗或雾化等处理方式比较，对术后咽喉疼痛的缓解快速有效，同时避免药物引起的相关不良反应。根据预实验结果表明此镇痛方式有一定优势，减轻患者术后疼痛，提高了患者舒适度及围术期疼痛管理质量。与传统的盲探方法比较，超声实时引导，定位准确，安全系数高，避免一些相关并发症的发生，超声引导下微创治疗手段也是日后疼痛治疗的发展趋势，值得临床研究及推广应用。

**二、研究开发内容、方法、技术路线**

1. **具体研究内容和重点解决的技术关键问题**

1、研究开发内容

本课题主要研究超声可视引导下喉上神经内侧支阻滞在治疗全麻拔管后咽喉疼痛的疗效，并与传统的雾化吸入比较，探索治疗全身麻醉气管插管术后咽喉疼痛的较理想的方案，为临床提供参考。

2、重点解决的技术关键问题

⑴ 操作技术层面问题：喉上神经部分在超声下成像不清楚，若合并解剖变异，有可能造成神经的损伤或是阻滞其它神经的可能。对操作者进行严格规范化培训，提高穿刺技术，设立准入制度，要求操作者熟练掌握解剖及超声基础，让超声实时引导的可视化可追踪发挥最大作用，避免了操作中相关不良反应的发生，初学者需在上级医师指导下进行；

⑵ 操作过程的无菌原则：严格消毒、铺无菌巾，探头使用无菌保护贴膜或保护套，置管后妥尚固定，术后及时随访观察穿刺部位的情况；

⑶ 严格入选标准:患者按择期禁食禁饮标准执行，避免反流误吸等的发生；操作前应得到患者的充分配合与理解，保持清醒，给药后应密切观察患者的生命体征的变化，严重者前耳鼻喉科会诊以排除严重的咽喉部损伤；

⑷ 及时随访观察不良反应的发生。

1. **项目的特色和创新之处**

1、喉上神经阻滞既往研究主要集中在在纤维支气管镜检查、喉内窥镜手术、困难气道清醒插管及辅助插管全麻等方面的应用，在单纯用于治疗气管插管全身麻醉拔管后咽喉痛方面的应用研究国内外尚未见报道，具有一定创新性；

2、本课题的研究在超声引导下进行双侧喉上神经内侧支阻滞，超声的可视化、实时性及精确性与传统的盲探方式比具有一定创新性，大大减少了不良反应的发生，提高了临床的安全性。

1. **采用的方法、技术路线以及工艺流程**
2. 资料与方法

1.1 一般资料：经医学伦理委员会批准，患者及其家属均签署知情同意书。选择本院全麻拔管后发生咽喉疼痛的妇科腹腔镜手术患者120例，年龄25～65岁，体重指数18～30 kg/m^２^，ASAⅠ或Ⅱ级。麻醉手术时间<4h,术后生命体征平稳患者120例，年龄18～65岁。 排除标准：（1）术前有长期咽喉部不适者或慢性咽喉炎患者；（2）对局麻药物过敏患者；（3）困难插管者及多次插管者；（4）有糖尿病或精神病病史患者；（5）长期接受阿片类药物、解热镇痛药物或激素治疗的患者。经医院伦理委员会同意并签署知情同意书后纳入本研究。采用电脑随机生成的数字法将患者随机分为利多卡因+布地耐德雾化组（L组）及喉上神经阻滞组（S组），每组60例。

1.2 方法

1.2.1 麻醉方式：所有患者（妇科腹腔镜手术）术前30 min肌注阿托品0.5mg。入室后开放静脉，予乳酸钠林格氏液8 ml/kg·h静脉滴注，常规监测BP、HR、SPO_2_。麻醉诱导给予舒芬太尼0.35μg/Kg，丙泊酚注射液 2mg/kg，顺式阿曲库铵 0.2mg/kg 静脉推注行气管插管(所有患者均一次插管成功，操作由同一高年资的麻醉医师完成)。术中采用NT监测麻醉深度，吸入七氟烷 2%～3%，间断推注舒芬太尼及顺式阿曲库铵维持麻醉，术中根据麻醉深度调整麻药用量。术毕，待患者自主呼吸恢复，潮气量及呼吸频率达到拔管标准，意识清醒后拔除气管导管，送麻醉恢复室（PACU）。

1.2.2 PACU期间观察处理：面罩40%吸氧，常规检查ECG、HR、BP、SpO2。根据咽喉疼痛的严重程度选择中到重度咽喉疼痛患者作为研究对象，随机分两组，每组60例。咽喉痛分级：0级：没有咽喉痛；1级：轻度咽喉痛(抱怨喉咙痛只有在通过询问发现)；2级：中等咽喉痛(个人主动抱怨喉咙痛)；3级：严重咽喉痛(严重疼痛，并出现声音的明显改变)。L组：2%利多卡因 100 mg（产家：中国大家制药有限公司，批号:6J85J2,20 mg/1ml）+布地奈德混悬液1mg（产家：AstraZeneca Pty Ltd，批号：H20140475,1mg/2ml）雾化吸入，15分钟内雾化完毕。S组：超声引导下行采用2%利多卡因 100 mg（5ml）双侧喉上神经阻滞组（每侧各50mg，2.5ml）。超声引导下喉上神经阻滞法：嘱患者去枕平卧，头部偏向对侧，8-13MHz高频线性探头纵向放置一侧下颌下区，采用平面外穿刺技术，在舌骨大角与甲状软骨之间进行扫查，可见甲状腺肌与甲状腺膜这两结构之间有一高回声团即为喉上神经，回吸无血，缓慢注入 2% 利多卡因2.5 mL 后拔针，局部压迫，观察 5 min，如无异常情况，以同法阻滞对侧。

1. 监测方法

⑴ 主要技术指标：记录两组治疗前即刻( T_0_) 、治疗后10 min( T_1_)、30 min( T_2_)、1h( T_3_)、2 h( T_4_)、4h(T_5_)、24h(T_6_)咽喉疼痛的视觉模拟评分(visual analogue scale，VAS)，0-10数字法表示疼痛程度（0为无痛，10为最剧烈的疼痛，由患者自己评分确定疼痛强度：1～3为轻度疼痛，4～6为中度疼痛，7～10为重度疼痛）。咽喉疼痛的缓解率：0度未缓解；I度为轻度缓解，疼痛评分下降1/4；Ⅱ度为疼痛评分下降1/2；III度为疼痛明显缓解，评分下降3/4；IV度为疼痛完全缓解，其中III度及Ⅳ度为镇痛显著有效。并根据声音嘶哑的严重性：0级：没有；1级：病人自诉声音嘶哑；2级：观察者听出明显声音嘶哑；3级：失音。

⑵次要技术指标：监测并记录患者上述时间点T_0_-T_6_MAP、HR及SPO2；观察反应发生情况：恶心、呕吐、窒息感、声嘶、呼吸困难及心动过缓等。并在咽喉痛治疗后2h嘱咐患者喝20ml水，观察有无呛咳及返流误吸的发生.

3. 统计学方法 采用SPSS 16．0 统计软件包对数据进行分析。计量资料以均数±标准差（±s）表示，组间采用t检验 、单因素方差分析（one-way ANOVA）方差分析进行统计学处理。计数资料以构成比或率表示，采用卡方检验。P<0.05认为差异有统计学意义。

3.研究技术路线图

1. **项目考核指标**
2. 发表核心论文2篇；
3. 经济考核指标：预计新增产值 54.00 万元 ，预计净利润 45.00 万元，预计出口创汇 3.00 万美元，预计税金总额 2.00 万元；
4. 预计提供科技或知识产权等服务情况：培训/讲座/咨询等服务数累计3 次。
5. **预期要达到经济指标及社会效益**

1、经济效益：具有良好的经济效益，每例可增收240元超声检查及引导费，以及300元左治疗费，预计每年可完成1000余例，创造约54万元/年，净利润45万/年的直接经济效益。

2、社会效益：超声引导下喉上神经阻滞治疗气管插管全麻术后咽喉疼痛大大提高了围术期疼痛管理质量，使镇痛效果得到保障，提高患者的满意度，为患者提供了舒适医疗服务，体现了其社会价值。同时也为学科和医院得发展做出贡献，赢得好口碑和声誉。

**三、工作基础和支撑条件**

**（一）承担单位概况（人员、资产、业务与管理状况）**

我单位是广东省第一家股份制民营三甲医院，具有独特的体制优势和一流的经营管理理念。重视专科建设，注重以人为本，推动医疗质量与安全。对于项目优先配置仪器设备，为优秀人才提供优厚待遇，对医师医疗、教学、科研工作及培训也长期给予经费投入。因此，医院为创新项目开展和管理提供相应的专项经费，能妥善解决有关经费问题。

**（二）本项目现有的研究工作基础（包括与本项目相关的****前期科研成果、团队建设、设备购置等）**

前期准备工作：我科室的学科带头人，长期以来致力于超声引导神经阻滞麻醉及镇痛的研究，并引进的先进技术，在科内准备教材、教案、教科书、模具等有计划、有步骤的开展对超声引导神经阻滞的规范化培训，并在临床带教实施，使项目的参与者熟练掌握基本操作。制定规范操作流程、编写著作，开展临床研究，加强对外互动交流。

前期科研成果：近年来，我科科研团队致力于超声可视化技术的研究，在学术交流及科研方面取得了较大的成果。发表相关的科研论文4篇，佛山市科技计划项目及佛山市卫生局计划项目共5项，举办国家级及省级超声可视化技术会议10余次，开办学习班近10次，即将启动编写著作，因此在此项目的研究方面具有厚实研究基础。
 设备购置：目前，我科已有便携式床边彩色多普勒超声仪四台、已备置相关书籍、光盘，和示教模具。随着业务的不断拓展，现有的仪器设备已远远不能满足临床需求，我们将再购进更多更高端更精密的仪器。

团队建设：我科科研团队强大，通过不断培训和学习，形成一支训练有素的学术及技术团对，能熟练掌握超声这项操作技术，无需引进外来技术人员。

**（三）本项目相关技术已获得的政府财政支持情况**

本项目暂未获得政府财政的相关支持，相关的立项获医院支持约5万。

**（四）对环境的影响及预防治理方案**

超声引导下喉上神经阻滞诊断为微创性操作，无任何辐射，不会直接对人体造成损害。床旁超声引导下有创操作较传统盲探法更加安全、并发症更少，只要提高操作的熟练程度，临床并无明显不良作用。需要注意的是穿刺部位感染。

防范措施：

注意操作过程的无菌原则，严格消毒、铺无菌巾，探头使用无菌保护贴膜或保护套，术后注意穿刺部位的观察。

**四、风险分析**

对项目的风险性及不确定因素进行识别，包括技术风险、人员风险、市场风险、政策风险及对环境的影响和预防治理方案等。

可能存在风险性和防范措施：

1. 侵入性操作可能造成神经损伤；
2. 颈部皮下组织较为疏松及神经走行变异，操作不当，可出现阻滞其它神经阻滞而出现不良反应；
3. 喉上神经部分在超声下成像不清楚，有可能出现治疗效果不佳情况；
4. 局部感染及反流误吸等相关并发症。

防范措施

1. 对操作者进行严格规范化培训，提高穿刺技术，设立准入制度，要求操作者熟练掌握解剖及超声基础；
2. 超声引导的可视化及可追踪性避免了操作中神经损伤等不良反应，初学者需在上级医师指导下进行；
3. 操作过程的无菌原则，严格消毒、铺无菌巾，探头使用无菌保护贴膜或保护套，置管后妥尚固定，术后及时随访观察穿刺部位的情况；
4. 严格入选标准，患者按择期禁食禁饮标准执行，避免反流误吸等的发生；
5. 操作前应得到患者的充分配合与理解，保持清醒，给药后应密切观察患者的生命体征的变化，严重者前耳鼻喉科会诊以排除严重的咽喉部损伤；
6. 及时随访观察不良反应的发生。

**五、本项目各参加单位工作分工及经费投入、支出情况**

本项目由佛山市禅城区中心医院有限公司独立完成，负责项目的选题与制定计划、项目的开展与实施、资料的整理与分析、项目的总结经费的投入情况：

| 经费支出 | 额度 | 用途说明 |
| --- | --- | --- |
| 设备费 | 5万 | 设备的维修与折旧费 |
| 材料费 | 0.2万 | 研究项目的耗材及教学书籍购买，医学教学软件的购置等 |
| 差旅费 | 1万 | 参加国内外会议 |
| 培训会议费 | 1万 | 举办会议及学习培训班 |
| 国际合作及交流费 | 1万 | 参加国际会议及到国外著名单位访问学习 |
| 出版/文献/知识产权事务费 | 1.3万 | 成果鉴定，申报评级及文章的发表版面费 |
| 人员费 | 0.1万 | 技术人员的培养及项目研究过程中投入的人力资源 |
| 专家咨询费 | 0.2万 | 与国内外专家保持沟通及技术的交流 |
| 项目管理费 | 0.2万 | 项目主管机构及辅助管理人员支 |
| 共计 | 10万 |  |

**六、以往承担项目完成情况及主要成果（近五年内）**

1. **承担国家省部级有关课题完成情况**

暂无

1. **以往科技成果转化情况**

近几年我科通过我科人员通过强有力的规范化培训，成就的一批专业化水准的人才队伍。我科曾多次主办围术期超声学术会议，开展学习班，成果斐然。现超声引导神经阻滞已被用于临床术前诊断、术中麻醉与监测、术后镇痛等多个领域，大大提高了诊治水平，推动了医学事业的发展。

1. **项目获奖及已发表的与本课题研究有关的主要论文、专著情况**

**相关课题**

1. 李朝晖,陈嘉华,何妹仪,等. 超声引导持续股神经阻滞在TKA术后镇痛及功能锻炼的应用.2013-2015.佛山市科技创新项目,201308126.

2. 王美容，何月贞，蒋群锰，李志鹏，王景泉，柳垂亮。自动定量输注法与持续输注法在TKA术后股神经旁置管镇痛中的效果比较，佛山市卫生局医学科研项目（2014178），2014.1-2015.12。

3. 何妹仪,何月贞,陈碧艳,柳垂亮,龚文魁,王美容,杨钧,陈东阳.超声引导胸椎旁神经阻滞在乳腺肿物切除术中的应用.2014-2016 佛山市科技创新项目. 项目编号2014AB001417.

4. 柳垂亮,蔡建历,蒋群锰,陈冬阳,李志鹏. 床旁超声与C型臂X光机引导选择性腰神经根阻滞的比较研究. 2015.1-2016.12. 2015年佛山市医学科研基金项目(2015133).

5. 柳垂亮,王美容,宋文平,何妹仪,陈太新,蒋群锰.脉冲式自动泵注与持续性恒速输注在超声引导下胸椎旁阻滞镇痛中的应用效果比较.2017.1-2018-12, 佛山市科技创新项目.项目编号2016ABOO2801.

6. 王美容,欧阳惠碧,林逸诚,李志鹏,何妹仪,柳垂亮.超声引导下喉上神经阻滞治疗气管插管全麻术后咽喉疼痛的效果评估.佛山市科技创新项目.项目编号 2017AB002411.2018-1至2019-12.

7. 陈太新,王美容,柳垂亮,龚文魁,李志鹏,蒋群锰.超声引导下持续收肌管阻滞与持续股神经阻滞在TKA术后的镇痛效果比较.佛山市卫生和计生局医学科研课题.项目编号:20180170. 2018-1至2019-12.

8. N0001 围麻醉期可视化技术的研究及推广 医院人才启动项目 2012-2014 5万元

**完成相关论文12篇**

1．陈太新,李志鹏,柳垂亮.超声法评价右颈内静脉走向的体表搏动目测法的准确性.医学信息,2015,1(2) :91.

2.王美容,何妹仪,李志鹏,柳垂亮. 自动定量输注法与持续输注法在TKA术后股神经旁置管镇痛中的应用效果比较. 广东医学,2016,37(17):2614-2616.

3.龚文魁,王美容,贾振华,何妹仪,柳垂亮. 超声引导下高位股神经阻滞在高龄患者腰硬联合麻醉前体位变动中镇痛效果研究.临床急诊杂志,2016,17(11):863-865.

4.李志鹏,何月贞,龚文魁,柳垂亮Co. 超声引导银质针治疗腰椎术后失败综合征的效果[J]. 广东医学,2017,38(6):872-874.

5.蒋群锰,李志鹏,王美容,杨钧,柳垂亮Co. 组织胶水减少神经置管术后漏液渗液的回顾性分析[J]. 实用疼痛学杂志, 2017, 13(2):116-119.

6.王美容,李志鹏,蒋群锰,龚文魁,陈太新,柳垂亮Co. 右美托咪定配伍罗哌卡因单次坐骨神经阻滞对术后股神经旁置管镇痛效果的影响[J]. 实用医学杂志, 2017, 33(7):1145-1148.

7.何月贞,李志鹏,柳垂亮,王美容,何妹仪. 肋间神经阻滞与胸椎旁阻滞对于乳腺肿物手术麻醉效果的比较. 广东医学,2017,38(24):3812-3815.

8.王美容,柳垂亮,蒋群锰,陈太新,何妹仪,蔡建历.脉冲式自动泵注与持续性恒速输注方式在超声引导下胸椎旁阻滞镇痛中的应用效果比较.广东医学,2017,38(23):3636-3640.

9.柳垂亮,王美容,蒋群锰,陈太新,何妹仪,程仕宇. 探讨开胸肺叶切除术后罗哌卡因胸椎旁脉冲式神经阻滞镇痛的适宜浓度. 实用疼痛学杂志,2018,14(1):46-51.

10.何妹仪,李志鹏,周健,王美容,柳垂亮. 围麻醉期床旁超声可视化技术的教学体会. 中国高等医学教育,2018,2:101-102.

11.王景泉,李志鹏,陆勇祥,柳垂亮.便携式超声仪车架的改良与应用. 中国医学装备,2013,10(12) :50-51.

12.陈嘉华,黄凯,李朝晖,等. 超声引导持续股神经阻滞在TKA术后镇痛及功能锻炼的应用[J]. 实用骨科杂志, 2015, 21(2):115-117.

**举办会议及学习班9期**

1. 围麻醉期超声技术学习班 2013 省级

2. 围麻醉期超声技术学习班 2014 省级

3. 精确麻醉实用新技术培训班2014 市级

4. 围麻醉期超声技术学习班 2015 省级

5. 精准靶向麻醉技术研讨会 2015 市级

6. 围术期床旁超声技术应用进展研讨会2016 国家级

7. 超声可视化技术应用研讨会2016 省级

8. 精准麻醉技术应用研讨会 2016 市级

9. 麻醉期床旁超声技术应用进展研讨会2017 国家级

10. 禅城区金马大型学术论坛 2017 市级

11. 超声可视化技术在麻醉和镇痛诊疗中应用进展2017 省级

12. 精准麻醉新进展培训班 2017 市级

**待启动编写著作一本。**
