# Supplementary material for: Ultrasound-guided internal branch of superior laryngeal nerve block on postoperative sore throat: A randomized controlled trial
Source: PLoS One. 2020 Nov 20;15(11):e0241834. doi: 10.1371/journal.pone.0241834 (PMC7679017; doi:10.1371/journal.pone.0241834)
Supplement: S2 Protocol — (DOCX) [file pone.0241834.s005.docx]

**The study protocol of ultrasound-guided internal branch of superior laryngeal nerve block on postoperative sore throat**

**（English edition）**

**1. Basis information** **of project establishment**

**1.1 Significance and objectives of the research**

Tracheal intubation is the main cause of postoperative sore throat (POS) caused by injury of the oropharynx or respiratory tract mucosa after general anesthesia. The main research purpose of this study is to explore the treatment of POST after general anesthesia and extubation. The aim of ultrasound-guided medial branch of the superior laryngeal nerve is to treat throat pain after general anesthesia extubation and provide a reference for reducing the incidence of POST after general anesthesia extubation. The main goal of the project: Through the development and research of this project, provide a good analgesic program for the treatment of sore throat after general anesthesia and tracheal intubation, improve the comfort and satisfaction of patients during perioperative period, and promote the development of anesthesia.

**1.2 Current status of similar products and technologies**

Postoperative sore throat (POST) is one of the common complications after tracheal intubation and general anesthesia. Studies have shown that the incidence of sore throat in patients with general anesthesia after extubation is 30% to 70%, mainly related to throat or respiratory mucosal damage. Although POST is self-healing, it will increase patient discomfort and prolong hospital stay, so it is still regarded as one of the unsatisfactory adverse reactions after general anesthesia. The occurrence of POST is related to many factors, such as tracheal tube factors, including its type and cuff pressure; operating factors, tracheal mucosal injury during intubation, etc.; surgical factors, including surgical time, posture, and type of surgery; in addition, women with a history of smoking is also a high risk factor for POST. With the deepening of the painless and comfortable medical model and the concept of rapid rehabilitation, medical workers are paying more and more attention to the prevention and treatment of POST. For patients undergoing general anesthesia surgery, POST is still an important problem to be solved.

Traditional treatment methods include drug treatment, aerosol inhalation, and application of local anesthetic drug cream at the end of the tracheal tube. Although it can alleviate a part of POST, it does not achieve the ideal effect, especially in patients with severe pain. Studies have shown that bilateral superior laryngeal nerve block can effectively suppress the stress response during extubation and reduce the occurrence of cardiovascular adverse events during tracheal extubation and anesthesia recovery. The supra-larynx nerve branch penetrates the thyroid hyoid membrane into the larynx, and is divided into many small branches to the pharynx, epiglottis, piriform crypt, and larynx mucosa above the glottis. The anesthesia effect of the larynx mucosa. It has been studied that superior laryngeal nerve block can better suppress hemodynamic changes caused by airway irritation during fiberoptic bronchoscopy, laryngoscope endoscopy, difficult airway intubation and auxiliary intubation general anesthesia. Provide better conditions for operation. Some studies on the effect of bilateral branch of the supra-larynx nerve in assisting laryngoscope surgery and evaluation of the impact on the incidence and severity of sore throat after general anesthesia indicate that the supraservical nerve's internal branch block has been relieved. The incidence of postoperative sore throat reduces the degree of postoperative sore throat, but it is rare in the research used for the treatment of postoperative throat pain alone, and no relevant reports have been reported in China.

**1.3 Development trend and forecast**

The internal branch block of the superior laryngeal nerve is ideal for the treatment of postoperative throat pain. Compared with traditional treatment methods such as drug treatment or nebulization, it can quickly and effectively relieve postoperative throat pain, and at the same time avoid related adverse reactions caused by drugs. The pre-experimental results show that this analgesic method has certain advantages, reducing patients' postoperative pain, improving patient comfort and perioperative pain management quality. Compared with the traditional blind detection method, ultrasound real-time guidance, accurate positioning, high safety factor, to avoid the occurrence of some related complications, ultrasound-guided minimally invasive treatment methods are also the development trend of pain treatment in the future, worthy of clinical research and popularization and application.

**2. Research and development contents, methods and technical routes**

2.1 Specific research content and key technical issues to be solved

This subject focuses on the efficacy of ultrasound-guided medial branch of the superior laryngeal nerve in the treatment of throat pain after general anesthesia extubation. Compared with traditional nebulized inhalation, it explores the treatment of throat pain after endotracheal intubation under general anesthesia. The ideal plan provides a ideal treatment for the clinic.

Operational technical issues: The imaging of the superior laryngeal nerve is not clear under ultrasound. If combined with anatomical variation, it may cause nerve damage or the possibility of blocking other nerves. We conduct strict and standardized training for operators, improve puncture technology, and establish an access system. Operators are required to master the basics of anatomy and ultrasound, so that the visualization of ultrasound real-time guidance can be traced to the greatest extent, avoiding the occurrence of related adverse reactions during the operation. Scholars need to conduct under the guidance of superior doctors.

2.2 The aseptic principle of the operation process: strictly sterilize, lay sterile towels, use a sterile protective film or protective sleeve for the probe, secure it after placing the tube, and follow-up and observe the puncture site in time after surgery.

2.3 Strict selection criteria: patients should be implemented according to the standard of fasting and drinking in order to avoid the occurrence of reflux and aspiration; the patient should be fully cooperated and understood before the operation, keep awake, and observe the changes in the patient's vital signs after administration , In severe cases, consult with the former ENT department to rule out serious throat injury.

2.4 Follow up to observe the occurrence of adverse reactions.

**3. Materials and methods**

3.1 General information: With the approval of the Medical Ethics Committee, patients and their families have signed an informed consent. 120 patients with gynecological laparoscopic surgery who had throat pain after extubation under general anesthesia in our hospital were selected. They were 25 to 65 years old, and had a body mass index of 18 to 30 kg/m2. Anesthesia operation time <4h, 120 patients with stable vital signs after operation, aged 18 to 65 years. Exclusion criteria: (1) Patients with long-term throat discomfort or chronic pharyngitis before surgery; (2) Patients allergic to local anesthetic drugs; (3) Difficult intubation and multiple intubation; (4) Diabetes or Patients with a history of mental illness; (5) Patients who have been receiving opioids, antipyretic analgesics or hormone therapy for a long time. It was included in this study with the consent of the hospital ethics committee and the signing of informed consent. The patients were randomly divided into a lidocaine + budesonide nebulization group (L group) and a supralarynx nerve block group (S group) by a computer-generated number method, with 60 cases in each group. The allocation of participants was performed by an independent researcher at each clinical site who was not involved in outcome assessment. Patients in both groups were treated in the postanesthesia care unit and blinded to which treatment they would receive. The outcome assessors, data collectors, and statisticians were blinded to group allocations during the study

**3.2 Major drugs and equipment**

2% Lidocaine hydrochloride injection: Batch No.: 6J85J2, 20 mg/1ml, Sandoz (China) Pharmaceutical Co., Ltd.

Budesonide suspension: Batch No.: H20140475, 1mg/2ml, AstraZeneca Pty Ltd.

Portable two-dimensional ultrasound instrument: icro Maxx HFL386, Sonosite Corporation, USA

Ultrasonic probe: Linear-array probe frequency of 6~1 3MHz, Sonosite Corporation, USA

Disposable medical atomizer (mouth-containing): Batch No.: 18050605, Excellentcare (Huizhou) Medical Ltd.

**4 Method**

**4.1 Anesthesia method:**

The study was approved by the Medical Ethics Committee of the hospital, the ethics committee approved before the trial began, and informed consent was signed by patients and their families. The register number was ChiCTR1800015007.

Patients were enrolled in the study from June 12, 2018, to June 6, 2019.

All patients (gynecological laparoscopic surgery) received intramuscular injection of 0.5 mg atropine 30 minutes before surgery. After entering the room, the vein was opened, and intravenous infusion of sodium lactate Ringer's solution 8 ml/kg·h was performed, and BP, HR, and SPO2 were routinely monitored. Anesthesia induction was given sufentanil 0.35μg/Kg, propofol injection 2mg/kg, cis atracurium 0.2mg/kg intravenous bolus intubation (all patients were successfully intubated at one time, the operation was performed by the same Senior anaesthetist completed). During the operation, Nacro Trend was used to monitor the depth of anesthesia, sevoflurane was inhaled 2% to 3%, sufentanil and cis-atracurium were intermittently injected to maintain anesthesia, and the amount of anesthetic was adjusted according to the depth of anesthesia during the operation. After the operation, the patient's spontaneous breathing recovered, the tidal volume and respiratory rate reached the standard of extubation, and the tracheal catheter was removed after being conscious, and sent to the anesthesia recovery room (PACU).

**4.2 Observation and treatment during PACU:**

Face mask 40% oxygen absorption, routine examination of ECG, HR, BP, SpO2. According to the severity of throat pain, patients with moderate to severe throat pain were selected as the research object, and randomly divided into two groups, 60 cases in each group. Sore throat grade: 0: no throat pain; 1: throat ache (complaining about sore throat only when found through inquiries); 2: throat throat moderate (personally complaining of sore throat); Grade 3: severe throat pain ( Severe pain, and obvious changes in voice). Group L: 2% lidocaine 100 mg (producer: China National Pharmaceutical Co., Ltd., batch number: 6J85J2, 20 mg/1ml) + budesonide suspension 1 mg (producer: AstraZeneca Pty Ltd, batch number: H20140475, 1 mg /2ml) Nebulized inhalation, complete within 15 minutes. Group S: Ultrasound-guided 2% lidocaine 100 mg (5 ml) bilateral superior laryngeal nerve block group (50 mg, 2.5 ml on each side). Ultrasound-guided superior laryngeal nerve block method: instruct the patient to lie down and lie on the head, the head is biased to the opposite side, 8-13MHz high-frequency linear probe is placed longitudinally on one side of the submandibular region, using out-of-plane puncture technology, at the large angle of the hyoid bone and the thyroid Scanning between the cartilage showed that there was a hyperechoic mass between the two structures of thyroid muscle and thyroid membrane, namely the supragaryngeal nerve, which was sucked back without blood. Slowly inject 2% lidocaine 2.5 mL and withdraw the needle, local compression, observe 5 min, if there is no abnormal situation, block the contralateral side by the same method.

**4.3. Monitoring methods**

**4.3.1** Main technical indicators: record the throat immediately before treatment (T0), 10 min (T1), 30 min (T2), 1h (T3), 2h (T4), 4h (T5), 24h (T6) of the two groups after treatment Visual analogue scale (VAS) of pain, 0-10 digital method indicates the degree of pain (0 is painless, 10 is the most severe pain, the pain intensity is determined by the patient's own score: 1 to 3 is mild pain, 4 to 6 are moderate pain, 7 to 10 are severe pain). Relief rate of throat pain: 0 degree is not relieved; I degree is mild relief, pain score drops by 1/4; II degree is pain score drop by 1/2; III degree is obvious pain relief, score drops by 3/4; IV degree For complete pain relief, III and IV are effective for analgesia. And according to the severity of hoarseness: level 0: no; level 1: the patient complained of hoarseness; level 2: the observer heard obvious hoarseness; level 3: aphonia.

**4.3.2** Secondary technical indicators: monitor and record the patient's T0-T6MAP, HR and SPO2 at the above time points; observe the reaction: nausea, vomiting, suffocation, hoarseness, dyspnea and bradycardia. And instructed the patient to drink 20ml of water 2h after the treatment of sore throat, to observe the occurrence of choking and reflux aspiration.

**5 Statistical analysis**

Statistical analysis was carried out by software package SPSS IBM 22.0, ages,intubation time, patient satisfaction score, VAS score of sore throat, blood pressure and heart rate were all analyzed in measurement data expressed as mean±standard deviation. Among them, age, inter-group comparison of BMI, intubation time and patient satisfaction score was achieved and analysed by t test; while inter-group comparison of VAS score of sore throat, blood pressure and heart rate was performed by variance analysis of repeated measurements. Greenhouse-Geisser's correction was applied when the Mauchly's test of sphericity was not met, and multivariate analysis of variance was used to compare Group L and Group S at each time point. Furthermore, gender, ASA grading and sore throat grade of the two groups were analyzed based on counting data using rate or percentage. Chi-square test was used to compare gender, ASA grading and analgesic efficacy between groups, and rank sum test was used to compare sore throat grading between groups. A P value of <0.05 was considered to be statistically significant. All data of this study will be uploaded to the website：http://www.chictr.org.cn/index.aspx.

**6. Risk analysis**

Identify the risks and uncertainties of the project, including technical risks, personnel risks, market risks, policy risks, impact on the environment and preventive governance plans.

There may be risks and preventive measures:

6.1. Invasive operation may cause nerve damage;

6.2. The subcutaneous tissue of the neck is relatively loose and the nerve runs are mutated. Improper operation may block other nerves and cause adverse reactions;

6.3 The imaging of the superior laryngeal nerve part under ultrasound is unclear, and there may be cases of poor treatment effect;

6.4. Related complications such as local infection and reflux aspiration.

**7 Precautions**

7.1 Conduct strict and standardized training for operators, improve puncture technology, and establish an access system, requiring operators to be proficient in the basics of anatomy and ultrasound;

7.2 The visualization and traceability of ultrasound guidance avoids adverse reactions such as nerve damage during operation. Beginners need to perform under the guidance of superior doctors.

7.3 The aseptic principle of the operation process, strictly sterilize and lay sterile towels, the probe uses a sterile protective film or protective sleeve, the tube is properly fixed after the tube is placed, and the puncture site is observed in time after the operation;

7.4 Strict selection criteria, patients should be implemented according to the standard of fasting and drinking, to avoid the occurrence of reflux aspiration;

7.5 The patient should be fully coordinated and understood before the operation, and keep awake. After administration, the patient's vital signs should be closely observed. In severe cases, the frontal otolaryngology consultation will rule out serious throat injury;

7.6 Follow up in time to observe the occurrence of adverse reactions.
