# Supplementary material for: Ultrasound-guided internal branch of superior laryngeal nerve block on postoperative sore throat: A randomized controlled trial
Source: PLoS One. 2020 Nov 20;15(11):e0241834. doi: 10.1371/journal.pone.0241834 (PMC7679017; doi:10.1371/journal.pone.0241834)
Supplement: Attachment — Submitted filename: Response to Reviewers.docx [file pone.0241834.s006.docx]

**1) Please ensure that your manuscript meets PLOS ONE's style requirements, including those for file naming. The PLOS ONE style templates can be found at**

<https://journals.plos.org/plosone/s/file?id=wjVg/PLOSOne_formatting_sample_main_body.pdf> and

<https://journals.plos.org/plosone/s/file?id=ba62/PLOSOne_formatting_sample_title_authors_affiliations.pdf>

**Dear, Editor，****We have change the style of article and file name to meet PLOS ONE's style requirements. You can see the details in the revised article. We also change the file name about the article.**

2) Thank you for submitting your clinical trial to PLOS ONE and for providing the name of the registry and the registration number. The information in the registry entry suggests that **your trial was registered after patient recruitment began**. PLOS ONE strongly encourages authors to register all trials **before recruiting the first participant in a study.**

As per the journal’s editorial policy, please include in the Methods section of your paper:

(a) your reasons for your delay in registering this study (after enrolment of participants started);

**In fact , we register on Feburary 28，2018,and recruited patients from June 12, 2018, to June 6, 2019.** You can find the details on [http://www.chictr.org.cn/searchproj.aspx by searching ChiCTR1800015007](http://www.chictr.org.cn/searchproj.aspx%20by%20searching%20ChiCTR1800015007).1

**In our protocol ，the time schedule is different because the preliminary experiment was made to make sure the technique is safe and useful, we apply to registered this study after that.**

**The authors confirm that all ongoing and related trials for this study are registered.**

Please also ensure you report the **date at which the ethics committee approved the study** as well as the complete date range for patient recruitment and follow-up in the Methods section of your manuscript."

**The details has been added in the article. We report the date at which the ethics committee approved the study as well as the complete date range for patient recruitment and follow-up in the Methods section.** **The ethics committee approved the study on Feburary 7，2018.**

**Because the syndrome is always self-healing， we follow-up the patients in 3days.**
3) Please provide additional details regarding **participant consent**. In the ethics statement in the Methods and online submission information, please ensure that you have specified (1) whether consent was informed and (2) what type you obtained (for instance, written or verbal, and if verbal, how it was documented and witnessed). If your study included minors, state whether you obtained consent from parents or guardians.

**The details has been added in the article.** **All patients gave written informed consent before participation in this study.**

4) In your Methods section, please provide additional information about the participant recruitment method and the demographic details of your participants. Please ensure you have provided sufficient details to replicate the analyses such as: a) a description of how participants were recruited, and b) descriptions of where participants were recruited and where the research took place.

Participants were recruited for the study through advisement from Chancheng central hospital and the research was took place in PACU.

5) Please provide **a sample size and power calculation** in the Methods, or discuss the reasons for not performing one before study initiation.

**We calculated that a sample size of 49 patients in each group would give the study 80% power to detect a 20-point greater improvement in VAS scores in the S group than in the I group at a two-sided significance level of 0.05.The calculation was operated on the website** **<http://powerandsamplesize.com.The> previous study has found that 90% of patients recovered after ultrasound guided injection of Isln,and 67.7% patients recovered after inhalation，so the proportion was used to calculated the sample size.** ***http://powerandsamplesize.com/Calculators/Compare-2-Proportions/2-Sample-Equality?***

6) To comply with PLOS ONE submission guidelines, in your Methods section, please provide additional information regarding your statistical analyses, including the name and version of the specific statistical software used for the analysis. For more information on PLOS ONE's expectations for statistical reporting, please see <https://journals.plos.org/plosone/s/submission-guidelines.#loc-statistical-reporting.>

**The software named IBM SPSS statistics 24.0 was used for the analysis.**

7) Please amend either **the title on the online submission** form (via Edit Submission) or the title in the manuscript so that they are identical.

**We have changed the file names to make sure they are identical.**

8) Please include **captions** for your Supporting Information files at the end of your manuscript, and update any in-text citations to match accordingly. Please **see our Supporting Information guidelines** for more information: <http://journals.plos.org/plosone/s/supporting-information>.

**The supporting information files has been added to the bottom of the article, we have tried to revised the article follow the guideline.**

9) **We note that** you have indicated that data from this study are available upon request. PLOS only allows data to be available upon request if there are legal or ethical restrictions on sharing data publicly. For information on unacceptable **data access restrictions**, please see <http://journals.plos.org/plosone/s/data-availability#loc-unacceptable-data-access-restrictions.>

In your revised **cover letter,** please address the following prompts:

a) If there are ethical or legal restrictions on sharing a de-identified data set, please explain them in detail (e.g., data contain potentially identifying or sensitive patient information) and who has imposed them (e.g., an ethics committee). Please also provide contact information for a data access committee, ethics committee, or other institutional body to which data requests may be sent.

**The de-identified data sets has been uploaded this time following the guideline. Telephone number of** **ethics committee was 0757-827788715**

b) **If there are no restrictions, please upload the minimal anonymized data set necessary to replicate your study findings as either Supporting Information files or to a stable**, public repository **仓库知识库**and provide us with the relevant URLs, DOIs, or accession numbers. Please see <http://www.bmj.com/content/340/bmj.c181.long> for guidelines on how to de-identify and **prepare clinical data for publication**. For a list of acceptable *repositories*, 仓please see <http://journals.plos.org/plosone/s/data-availability#loc-recommended-repositories.>

**We uploaded** **de-identified data sets as supplyment materials this time following the guideline.**
